# Microfluidic platform for understanding Parkinson’s disease and α-synuclein conformation

**DOI:** 10.1186/s40035-025-00526-0

**Published:** 2025-12-24

**Authors:** Tregub Pavel, Zembatov Georgy, Namiot Eugenia, Kolotyeva Natalia, Yurchenko Stanislav, Illarioshkin Sergey, Salmina Alla

**Affiliations:** 1 Russian Center of Neurology and Neurosciences, Moscow, Russia 125367; 2 Bauman Moscow State Technical University, Moscow, Russia 105005; 3 Pathological Physiology Department, I.M. Sechenov First Moscow State Medical University (Sechenov University), Moscow, Russia 119991; 4 RUDN University, Moscow, Russia

**Keywords:** Microfluidic, Lab-on-a-chip, Parkinson’s disease, α-Synuclein, Aberrant protein, Organ-on-a-chip

## Abstract

Microfluidic systems are an innovative engineering solution that is increasingly being used in a wide range of scientific fields. These systems use fluids in microchannels (1 to 300 microns) to analyze extremely small volumes of sample and reagent, allowing precise delivery and mixing while maintaining accurate results. Parkinson's disease (PD) poses significant diagnostic challenges, with early detection being critical to improved treatment outcomes. A key pathological feature of PD is the presence of Lewy bodies composed of α-synuclein (αSyn) fibrils. Recent research has shown that αSyn oligomers can be toxic and contribute to neuronal loss. Therefore, microfluidics offers a promising approach for the diagnosis of different stages of αSyn pathology. This review comprehensively analyzes the application of microfluidics in single-cell analysis and protein aggregation studies. We discuss the concept of lab-on-a-chip analysis and examine different substrates for αSyn detection, citing relevant studies and expected protein concentrations and their correlations with disease progression and severity.

## Background

Parkinson’s disease (PD) is the second most common neurodegenerative disorder after Alzheimer’s disease, affecting approximately 1% of the population over 60 years of age, with rising prevalence due to global aging [1]. Clinically, PD is characterized by motor symptoms such as bradykinesia, resting tremor, rigidity, and postural instability, as well as non-motor manifestations including cognitive decline, sleep disturbances, and autonomic dysfunction [2, 3]. The pathological hallmarks of PD include the progressive loss of dopaminergic neurons in the substantia nigra pars compacta and the accumulation of misfolded α-synuclein (αSyn) aggregates in Lewy bodies and Lewy neurites [4, 5]. Despite advances in symptomatic treatments like *L*-DOPA and deep brain stimulation, the disease remains incurable, underscoring the need to understand its molecular mechanisms to develop etiological and pathogenetic therapies [6].

At the molecular level, PD is characterized by the presence of intraneuronal Lewy bodies composed of αSyn amyloid fibrils, which are also found in some other neurodegenerative diseases, such as the Lewy body variant of Alzheimer's disease [7–9].

Today, the diagnosis of PD is based on clinical features, post-mortem examination, and neuroimaging [3, 10, 11]. At the same time, it is known that signs of synucleopathy can be observed many years before the clinical manifestation of the disease and morphological manifestations [12]. Therefore, the detection of aberrant forms of αSyn in body fluids is a promising approach for the development of new methods for the early diagnosis of this disease.

**Fig. 1:**
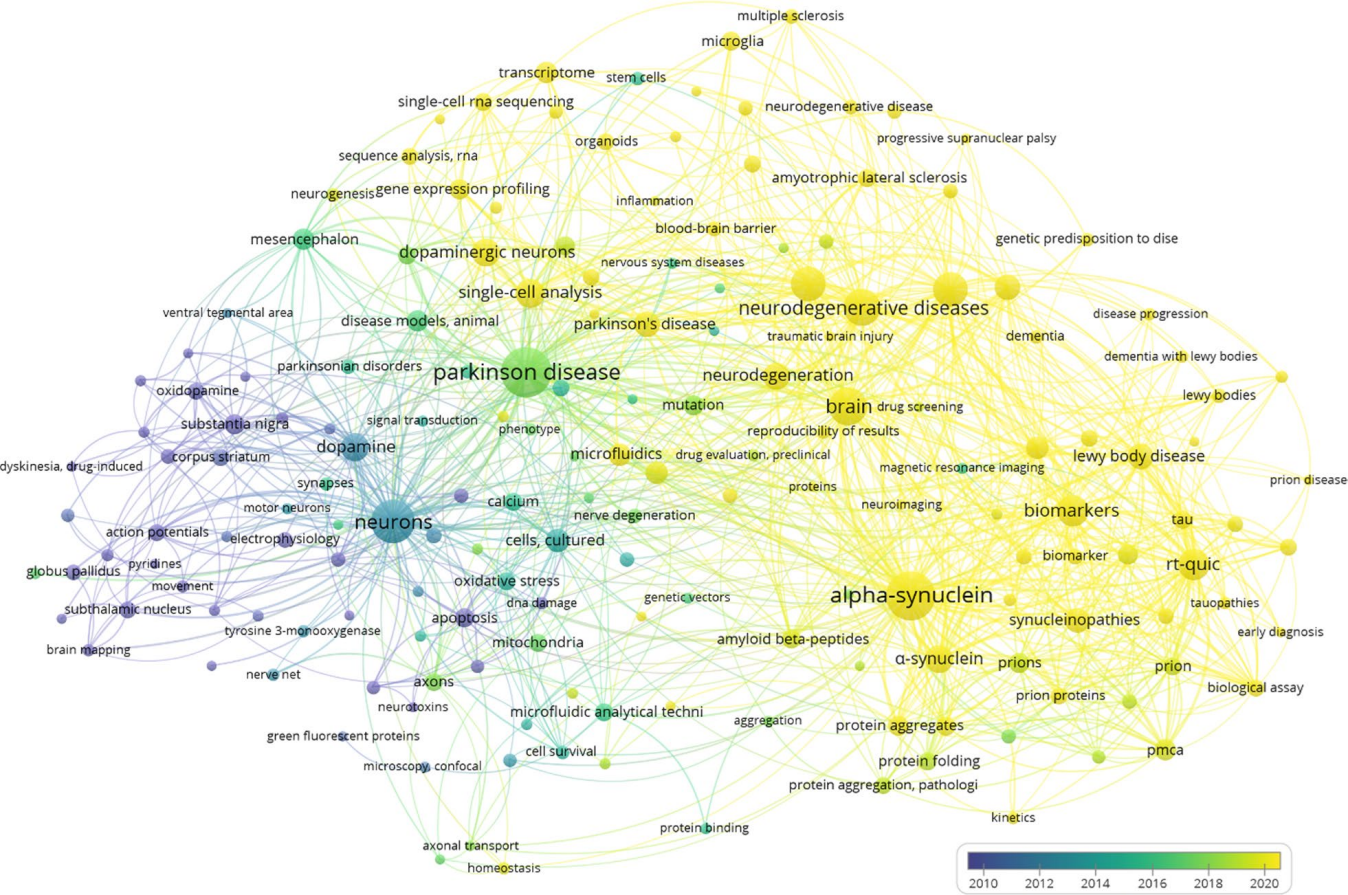
A diagram of the occurrence of keywords in PubMed database publications for the period 2005–2025 on the topic "microfluidic systems and synucleinopathies". The diagram was constructed using the VOSviewer v.1.6.20 program

Microfluidic systems, including lab-on-a-chip (LoC) and organ-on-a-chip (OoC), are among the most promising technologies in neuroscience for accurate protein analysis in basic research and for diagnostic applications. If we analyze the publications in the PubMed database for the previous 40 years (1984–2025), we can find 499 papers devoted to these areas (Fig. 1), with the majority (almost 68% or 339 papers) from the last 5 years. At the same time, the topics of these publications are most often related to studies of pathogenesis and diagnostic possibilities (including topical ones), associated with the concept of prion diseases, characteristics of methods, and related to the joint study of biomarkers of various neurodegenerative diseases, such as αSyn, tau protein, and beta-amyloid. Interestingly, over time, trends have become more focused on protein amplification techniques and epigenetics.

The aim of this review is to analyze the potential applications of microfluidic technologies in the study of aberrant proteins, with a particular focus on αSyn. The review will provide a detailed examination of the capabilities of microfluidic technologies to assess the aggregation of aberrant proteins and will analyze data on the use of microfluidic platforms to investigate the mechanisms underlying the pathogenesis of synucleinopathies. It will also evaluate the prospects and potential for expanding the use of microfluidic systems in research into the pathogenesis of neurodegenerative diseases and their diagnosis.

## αSyn overview

**Fig. 2:**
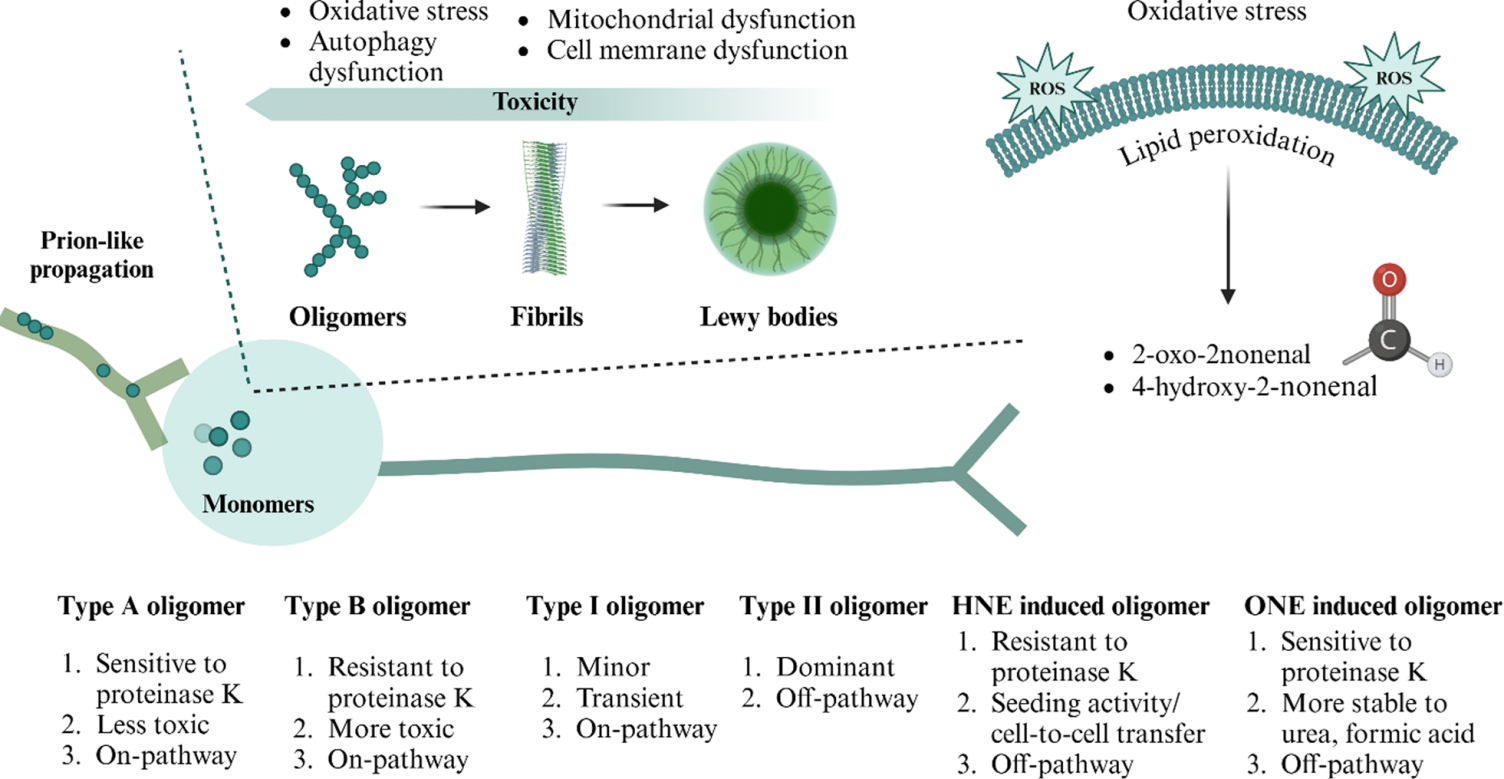
Types of αSyn oligomers and their toxic mechanisms. The different types of αSyn oligomers—Type I, Type II, Type A, and Type B—each play a pivotal role in the pathogenesis of neurodegenerative diseases, with their toxic mechanisms contributing significantly to disease progression. Type I oligomers are transient and less abundant, whereas Type II oligomers are more stable. Type A and B oligomers are distinguished based on their level of toxicity, with Type B being considered more toxic due to its greater ability to induce cellular damage [14]. These oligomers are involved in the formation of mature αSyn fibrils, which contribute to the development of Lewy bodies, and may also propagate in a prion-like manner [225, 226]. Among these forms, the most toxic are the oligomers, which induce mitochondrial damage, adhere to cell membranes, increase membrane permeability, and stimulate oxidative stress [101, 225]

αSyn oligomers play a crucial role in the pathogenesis of neurodegenerative diseases, with different types contributing to disease progression through distinct mechanisms [13, 14]. These oligomers can be broadly categorized into structural and functional types, including Type I, Type II, Type A, and Type B, varying in stability and toxicity. Type I oligomers are transient and less abundant, while Type II are more stable. Types A and B differ in toxicity, with Type B being more damaging to cells [15]. Type I oligomers are notable for their propensity to form fibrils, while Type B oligomers are characterized by their resistance to proteinase K. This resistance is thought to stem from a unique epitope present in the αSyn sequence of Type B oligomers, which could be exploited for the generation of antibodies with high specificity toward this oligomeric form [13, 14]. Additionally, αSyn oligomers can be classified based on molecular weight, encompassing monomers (15 kDa), low-molecular-weight oligomers (30–160 kDa), and high-molecular-weight oligomers (≥ 260 kDa) [16]. High-molecular-weight oligomers stimulate oxidative stress, induce apoptosis, and disrupt membrane functionality by integrating into the lipid bilayer, even when present in extracellular spaces [17, 18]. While off-pathway oligomers, which do not contribute to fibril formation, remain unconfirmed, their potential toxicity warrants further investigation, as they may evade protective mechanisms such as Lewy body formation [19]. The main types of αSyn oligomers and their mechanisms of toxicity are illustrated in Fig. 2.

Oxidative stress, a hallmark of many neurodegenerative diseases including PD, exacerbates cellular damage, particularly through peroxidative injury to membrane lipids [20, 21]. The by-products of lipid oxidation, such as 4-hydroxy-2-nonenal (HNE) and 4-oxo-2-nonenal (ONE), not only have cytotoxic effects but also promote the formation of specific αSyn oligomers [22]. HNE-induced oligomers, characterized by larger size and structured β-sheet configuration, exhibit seeding activity and can facilitate intercellular transmission. αSyn seeding activity refers to the ability of misfolded αSyn aggregates to induce the misfolding of normal αSyn molecules in vitro, forming the basis of amplification assays such as real-time quaking-induced conversion (RT-QuIC) and protein misfolding cyclic amplification (PMCA). Because this property enables highly accurate detection of pathological αSyn, seeding activity has emerged as one of the most promising diagnostic biomarkers for PD. In a clinical cohort of 1123 participants, Siderowf et al. demonstrated that the assay achieved a sensitivity of 87.7% and a specificity of 96.3%, supporting its potential for real-world diagnostic use [23]. Despite their β-sheet structure and seeding-like properties, HNE-induced oligomers are generally regarded as off-pathway species, because they form stable assemblies that do not elongate into fibrils even after prolonged incubation [24, 25]. Notably, HNE-induced oligomers are resistant to degradation by proteinase K, further distinguishing them from other forms [26]. Although the HNE-induced oligomers can act as toxic seeds in cellular systems, whether such aldehyde-induced oligomers contribute to fibril formation in vivo remains an open question under active investigation.

It is important to note that the most neurotoxic forms may not be mature amyloid fibrils, but rather soluble oligomers of αSyn and other intermediate structures that are detrimental to cells [19]. Protofibrils (prefibrillar oligomers) can inhibit proteasomes and increase vesicle permeability, leading to cellular damage even before aggregates form [27, 28]. Post-mortem analyses of brain samples from PD patients have also revealed extensive diffuse deposition of αSyn oligomers [29]. Accumulation of αSyn oligomers inhibits tubulin polymerization and disrupts the cytoskeleton [30]. Under normal circumstances, the body uses several systems to react to abnormal protein detection. A reduction in the activity of the autophagy system, combined with the stabilization of αSyn oligomers by dopamine, leads to the specific accumulation of aggregates in dopaminergic neurons [18, 19]. The newly formed αSyn oligomers disrupt axonal transport and impair mitochondrial function [19]. αSyn oligomers exist in various conformations, including ring-shaped, spherical, and tubular forms, which differ in their toxicity and interactions with cells [31–37]. The formation of these oligomers depends on factors like monomer availability, temperature, pH, and genetic mutations [38, 39].

In recent years, increasing attention has been paid to off-pathway oligomers—toxic αSyn species that do not proceed to form amyloid fibrils and may bypass classical aggregation pathways [40]. These oligomers often exhibit high β-sheet content and enhanced cytotoxicity, while remaining structurally stable and resistant to degradation. Their role in neurodegeneration is significant, as they have been shown to permeabilize cellular membranes [41] and interfere with mitochondrial function [42, 43]. Off-pathway species challenge the classical model that associates toxicity solely with fibrils or on-pathway intermediates.

This variability in oligomeric forms—ranging from spherical to annular and tubular shapes—demands advanced analytical tools capable of capturing transient, low-abundance, and structurally diverse protein species [31, 36, 37]. Microfluidic systems fulfill this need by offering precise control over physicochemical parameters such as pH, temperature, and ionic strength in confined volumes, enabling real-time tracking of oligomerization kinetics at single-molecule resolution [32, 38]. Furthermore, microfluidics can integrate advanced detection techniques—including fluorescence correlation spectroscopy (FCS), micro free-flow electrophoresis (μFFE), and aptamer-based sensors—which enable the isolation and quantification of distinct αSyn oligomer types under physiological conditions [44–46]. These capabilities make microfluidics uniquely positioned to dissect the structural heterogeneity and pathological significance of oligomeric αSyn species, which traditional bulk methods often fail to resolve due to averaging effects and low throughput [29, 47, 48].

## Microfluidic technology overview

Microfluidic systems represent a promising engineering solution that is being implemented in various fields of science. These systems are based on the use of fluids in microchannels (capillaries typically measuring between 1 to 300 μm) [49]. One of the main advantages of such systems is their ability to use extremely small amounts of samples and reagents while still providing accurate results. Another advantage of microfluidic systems is their ability to control small volumes of fluid, ensuring precise delivery and mixing of samples and reagents. Furthermore, these systems can process multiple samples simultaneously due to the presence of independent compartments [49–51]. A major advantage of microfluidic devices lies in their adaptability; the design is highly customizable, making them versatile tools for a wide range of applications [52]. Microfluidic technologies have already been successfully applied to the analysis of various biological samples, including DNA and proteins [51, 53].

A typical microfluidic system consists of several key components. The core of the system consists of microchannels (10 to 300 microns) etched in glass, silicon or polymers through which fluids flow. These channels are equipped with inlets and outlets that act as ports for the introduction of samples, reagents and buffers, as well as for the collection of processed materials. Micropumps and microvalves regulate the flow of fluids within these channels, directing them into different pathways or chambers [54, 55]. Integrated detection zones equipped with sensors (optical, electrical, etc.) monitor molecular substances within the system. For example, fluorescence detection zones are equipped with optical components to excite fluorescent labels on proteins and measure their emitted light, allowing real-time analytical data collection [56]. This integrated approach enables microfluidic systems to provide a controlled environment for protein analysis, which is useful for both research and diagnostic applications.

**Fig. 3:**
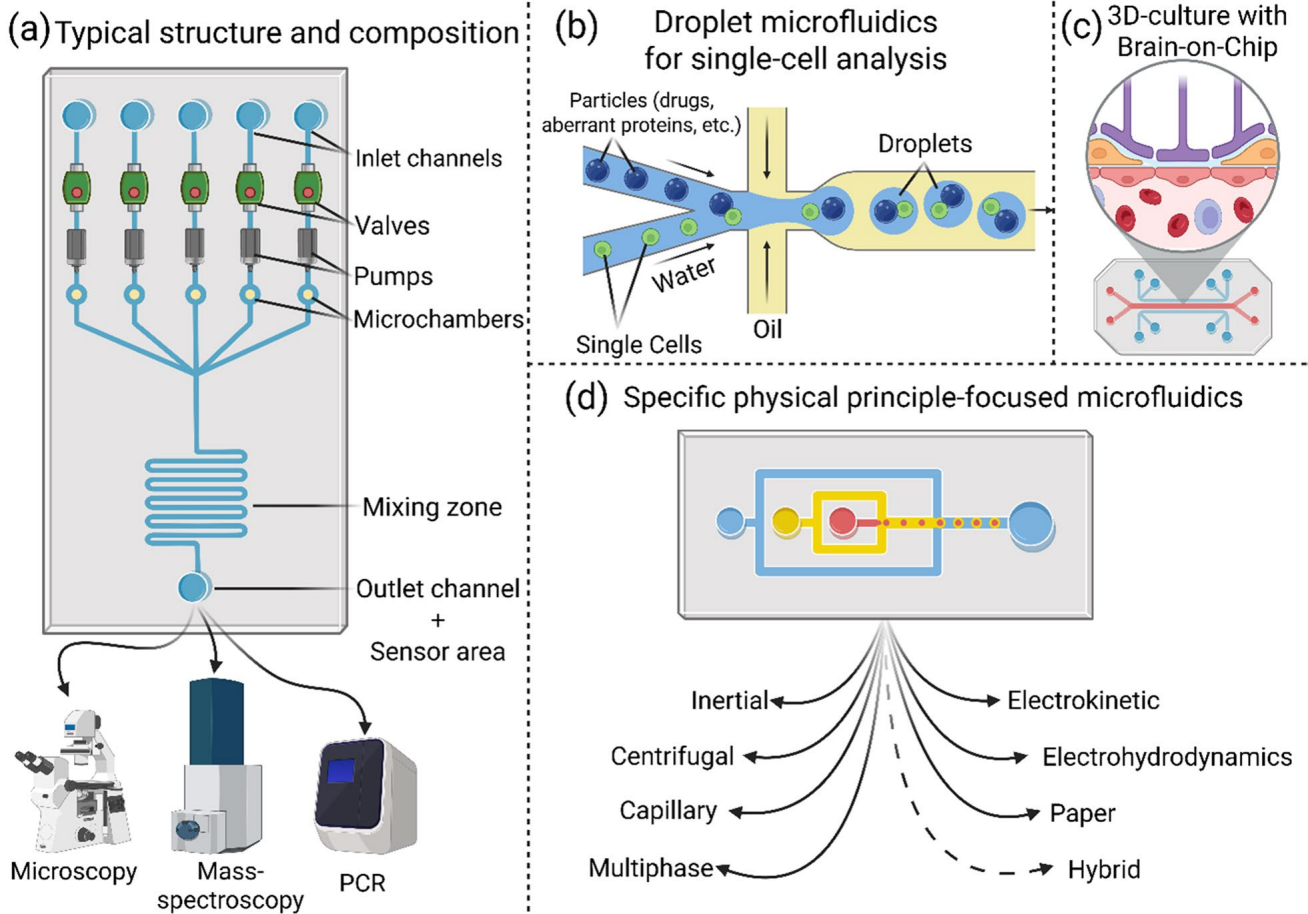
Overview of microfluidic device construction and types.** a** A typical microfluidic device consists of key components such as inlet and outlet regions, connected by microchannels. These microchannels can be tailored with additional features like valves, pumps, and microchambers, depending on the specific objectives of the study. In many applications, mixing zones are incorporated to facilitate biochemical reactions [52, 57–59]. The outlet region often integrates advanced detection techniques, with examples including MS, microscopy, and PCR, enabling precise analysis of the system's outputs [227–229]. **b** Among the most discussed microfluidic configurations is droplet microfluidics, which involves the manipulation of droplets, often formed by the interaction of water and oil. This technique is particularly valuable in single-cell research, a rapidly advancing area with significant implications for the study of neurodegenerative diseases [230, 231]. **c** Another notable variation is 3D culture microfluidics, where devices typically incorporate hydrogel layers, allowing for the creation of more complex structures such as brain-on-a-chip models [227]. These models are pivotal for studying cellular behaviors in a three-dimensional environment, closely mimicking in vivo conditions. **d** Microfluidic systems can also leverage various physical principles that govern particle movement within microchannels [92]. Microfluidic actuation spans inertial microfluidics (wall- and shear-gradient lift) [92], electrokinetic control (electric-field–driven transport) [93], centrifugal platforms (centrifugal forces) [232], electrohydrodynamics (field-driven manipulation of fluids and particles) [233], capillary and paper systems (capillarity in cellulose matrices) [135], multiphase microfluidics (handling multiple phases in one channel) [234], and hybrid approaches that combine these modalities[235].

Microfluidics focuses on systems designed to manipulate small volumes of fluids, leveraging principles of fluid dynamics and colloidal interactions at the microscale [57, 58]. At these dimensions, fluid flow is no longer predominantly governed by traditional pumps; instead, forces such as surface tension and capillary action become key drivers [59]. Capillary forces, in particular, enable fluid movement through narrow channels, exemplifying the precision of microfluidic control [52]. The essential components of a microfluidic system include microchannels, which can be tailored to specific research objectives, chambers for processes such as mixing, valves to regulate fluid flow, and sensor areas for monitoring and analysis [52, 60]. Fig. 3 illustrates the basic microfluidic device workflow.

### LoC technology

Microfluidics, as a multidisciplinary technology that manipulates fluids at the microscale, serves as the foundational platform for a wide range of biological and biomedical applications. Among its most prominent implementations are LoC devices, which integrate multiple laboratory functions—such as sample preparation, chemical reactions, and detection—on a single micro-scale chip. LoC systems inherently rely on microfluidic principles for fluid control and sample processing [24–26]. The size of these LoCs can vary from a few millimeters to several centimeters [24]. The primary motivation for the development of such chips is the need for rapid and accurate analysis that requires minimal input resources. Microfluidics plays a crucial role in this framework by enabling efficient manipulation and analysis of samples in microscopic volumes [24, 25]. In addition to microfluidic systems, LoC devices can incorporate amplifiers that enhance signals for improved detection, as well as biosensors that provide direct measurements of various parameters [61, 62]. These technologies are integrated to create compact and highly efficient analytical devices capable of performing complex laboratory tasks in a shorter time frame and at minimal cost. Over the last few decades, significant progress has been made in the development of LoC devices and micro total analysis system (μTAS) [54, 63].

The LoC technology shares similarities with microdevices for μTAS, as both work with small sample volumes [64]. However, the core principle of LoC is that all processes are integrated directly on the chip, while microfluidics can be a part of a broader system that may include external components for analysis, control, or detection. In contrast, LoC devices aim for complete miniaturization and automation of laboratory functions—such as mixing, separation, and detection—onto a single, compact platform. This distinction emphasizes the all-in-one nature of LoC systems, whereas microfluidic components may serve specialized functions within larger analytical setups. LoC allows high-throughput screening, which in turn enables the technology to be applied to the diagnosis of several diseases, e.g. HIV [65]. To successfully integrate microfluidic technologies into automated LoC systems, researchers are exploring several innovative approaches. Another such method is multilayer soft lithography, which uses polymer layers to create intricate planar multilayer microfluidic structures [66].

An example of a successful LoC implementation is the Triage protein chip developed by Biosite, a platform that enables the simultaneous measurement of over a hundred different proteins, demonstrating the practical potential of these advanced microfluidic technologies [67].

To fully realize the potential of these platforms, it is essential to consider the key components and processes that enable their functionality, from sample injection to analysis, within a LoC device. A standard LoC device consists of several key components, each designed to perform specific functions for sample processing and analysis. The process begins with an injector that introduces samples into the chip, typically using a syringe pump [68]. Once introduced, the sample may enter a preparation module where it undergoes various pre-processing steps such as filtration, cell lysis or electrophoretic separation [26, 68]. Throughout the chip, the sample is transported according to microfluidic principles, such as maintaining laminar flow, which ensures efficient fluid movement through the microchannels. The final component encompasses a detection device, which is chosen individually based on the study goal.

### OoC analysis

A further specialization of LoC is the OoC concept, which emulates the physiological responses of specific human organs using microfluidic structures and living cells. These systems are capable of mimicking the functional units of organs [69–71] by offering dynamic mechanical cues, flow environments, and tissue-tissue interfaces critical for modeling human biology more realistically than static culture models [72, 73]. For instance, brain-on-a-chip platforms recreate key components of the neural microenvironment, such as the blood–brain barrier and synaptic signaling [74].

These devices offer opportunities to model physiological and pathological processes under conditions that closely mimic the natural environment [69]. In essence, organs-on-chips represent a new generation of 3D culture models that overcome the limitations of their static predecessors, such as 2D cultures. Microfluidic platforms can replicate a dynamic microenvironment similar to that experienced by cells in vivo [75, 76], which is not possible with static macroscopic models. These microfluidic platforms can generate biochemical gradients of metabolites, soluble factors, cytokines and other molecules that affect the cellular microenvironment [77, 78]. As such, they allow more realistic and functional modeling of processes at the cellular and tissue level.

Bolognin et al. [16] have demonstrated the potential of using 3D microfluidic devices for modeling PD in vitro, advancing from traditional 2D cell cultures (Table 1). They showed that 3D cultures recapitulated time-dependent dopaminergic degeneration linked to the *LRRK2*-G2019S mutation, a common cause of familial PD. Kane et al. [79] developed the Pelican Organ-on-a-Chip system, integrating microfluidic cell culture with laboratory automation. This system automates the culture and differentiation of human neuroepithelial stem cells into dopaminergic neurons in a 3D microfluidic environment, closely mimicking in vivo conditions. Using the SiLA software for device communication, Pelican enables precise, scalable, and long-term monitoring of PD models [79] (Table 1).

**Table 1:** Key studies utilizing single-cell analysis technology and organ-on-a-chip platforms to investigate PD

Study	Year	Sample	Microfluidic technology	Main findings	Reference
Kane et al.	2020	iPSCs	OrganoPlate (#9603-200B)	Allowed for 24 h flow Optimized protocol for neuronal cell culture Demonstrated use of microfluidics for controlled cell culture and differentiation	[79]
Novak et al.	2022	hiPSCs (mDA neurons)	Drop-Seq (SC-RNAseq)	Identified pathways implicated in the early stages of PD including those involved in mitochondrial function Identified the presence of a common network of PD-associated pathways PD-associated pathways are dysregulated before any signs of disease	[90]
Perrino et al.	2019	Yeast cells	MFD0005a device	Microfluidics allows studies of αSyn aggregates in cells in real time Quantitatively investigated WT αSyn clearance	[257]
Bolognin et al.	2019	hNESC-derived neurons	OrganoPlates (Mimetas)	Microfluidic device for 3D neuron culture Genetic background, not the *LRRK2*-G2019S mutation, contributes to the PD phenotype	[16]
Brahic et al.	2016	Primary cortical neurons (mouse)	Xona SND 450	Microfluidics was used to study axonal transport Demonstrated prion-like properties of synuclein fibrils and quantified internalization	[97]
Tran et al.	2014	Primary hippocampal cultures (mouse)	Xona Microfluidic (TCND500)	αSyn antibodies blocked protein entry to the cells and its propagation Identified αSyn transmission as a central event in PD	[258]
Seidi et al.	2011	PC12 neuronal cell line	Custom (soft lithographic method)	Used 6-hydroxydopamine (6-OHDA) to study neuronal cell death in PD model At low concentrations of 6-OHDA along the gradient (i.e., approximately less than 260 μM), the neuronal death in the channel was mainly induced by apoptosis, while at higher concentrations, 6-OHDA induced neuronal death mainly through necrosis	[259]
Cavaliere et al.	2017	Primary cortical neurons (mouse)	Millipore (AXIS chambers)	Reported that astrocytes and neurons are able to uptake αSyn aggregates, which then induce cell death	[260]
Volpicelli-Daley et al.	2011	Primary hippocampal cultures (mouse)	Xona Microfluidics	Demonstrated that aggregates first appear in axons and then propagate through the rest of the cell, inducing cell death	[13]
Kamudzandu et al.	2019	Several neuronal subtypes cultures	Custom five-port microfluidic device (lithography methods)	Created a platform for multiple neuronal cultures to study PD and HD	[80]
Maisonneuve et al.	2021	Primary hippocampal cultures (mouse)	Custom two-chambered chip made of polydimethylsiloxane (PDMS)	Constructed basal ganglia loops to model PD and HD disease	[81]
Freundt et al.	2012	Primary cortical neurons (mouse)	Xona microfluidics	Microfluidics was used for separation of axon and soma Showcased anterograde transport of fibrils (fast axonal transport with saltatory movements) and explained anatomical pattern of PD distribution	[98]
Gribaudo et al.	2019	WT-human iPSCs (healthy donor)	Custom chip (PDMS)	Two distinct strains of αSyn (fibrils and ribbons) propagate in a prion-like manner in human neuronal networks, recruiting endogenous αSyn into toxic aggregates and causing Parkinson’s-like pathology, including synaptic and mitochondrial dysfunction. Ribbons were more potent than fibrils in seeding aggregation, highlighting conformation-dependent toxicity in sporadic PD	[95]
Prots et al.	2018	hiPSCs	Xona SND 450	Oligomers are responsible for early mitochondrial damage in synucleinopathies Early phenotype – disrupted axonal mitochondrial transport Oligomers are able to inhibit axonal transport	[96]
Fernandes et al.	2016	Human neuroglioma cells, microglial cells	Custom two-chambered device (PDMS)	Activated N9 microglial cells are able to increase ROS in cocultured H4 neuroglioma cells, showcasing neuroinflammation as part of PD progression	[91]
de Rus Jacquet et al.	2023	hiPSCs	OrganoPlate 3-lane 40	*LRRK2* G2019S mutant astrocytes are proinflammatory and impair BBB formation MEK1/2 inhibition reduces astrocyte inflammation and rescues BBB integrity	[261]
Pediaditakis et al.	2021	hiPSCs	Chip-S1	αSyn fibrils induce phosphorylated αSyn accumulation, mitochondrial impairment, and neuroinflammation Reproduced key PD features, including compromised BBB function	[262]

*iPSCs* Induced pluripotent stem cells, *hiPSCs* Human induced pluripotent stem cells, *mDA* Midbrain dopamine, *SC*-*RNAseq* Single-cell RNA sequencing, *PD* Parkinson’s disease, *6-OHDA* 6-hydroxydopamine, *BBB* Blood–brain barrier, *HD* Huntington’s disease, *PDMS *Polydimethylsiloxane, *αSyn* α-synuclein

Recent brain-on-a-chip studies have used the microfluidic technology to model the basal ganglia (BG) loop, which is crucial in PD. Kamudzandu et al. (2019) developed an in vitro brain-on-a-chip model with five compartments connected by unidirectional microchannels. They co-cultured specific neuronal subtypes from rodent brains, demonstrating direct connectivity across the neuronal circuit [80]. Similarly, Maisonneuve et al. created a brain-on-a-chip model of the BG loop, relevant to both Parkinson’s and Huntington’s diseases, using five interconnected compartments [81].

### Microfluidics for single-cell analysis

In the last decade, the importance of studying individual neurons has been increasingly highlighted as essential for understanding the development of pathologies and the overall functioning of the brain [82–84]. Standard methods that assess averages across cell cultures often fail to provide a complete understanding of individual cell behavior [85, 86]. Particular attention is being paid to modeling the function of individual axons, as this allows detailed exploration of their roles and interaction mechanisms. Microfluidic systems play a key role in this endeavor, allowing precise control of the microenvironment and conditions for the cells, thus improving our understanding of cellular processes at the level of individual neurons and axons [87]. However, when using single-cell analysis to study protein expression, a challenge is to accurately determine low levels of proteins both inside and outside the cell [88].

In the context of PD, single-cell analysis and the study of cell cultures are crucial to elucidate the molecular effects of αSyn and its aggregates. Current disease models inadequately capture the pathogenesis of the disease and often overlook the interactions between cells and their mutual influences [89]. In addition, it has been shown that the development of dopaminergic neurons in the midbrain differs from that of other dopaminergic neuron populations, which may complicate some complex brain-on-a-chip reconstruction systems [90]. Additional studies have highlighted the importance of intercellular interactions, such as the stimulation of aberrant αSyn folding by activated microglia, which is associated with the perpetuation of neuroinflammation [91]. Therefore, microfluidic systems that facilitate the study of individual cells and cell populations are excellent tools for modeling and studying PD.

Microfluidic systems can be used to diagnose diseases in living subjects, study post-mortem samples, and analyze neuronal cultures derived from stem cells, although cellular heterogeneity presents significant challenges in working with these cultures [92, 93]. Moreover, obtaining statistically significant analysis results requires working with multiple cells simultaneously, highlighting the importance of high-throughput analysis [94]. Patient-derived induced pluripotent stem cells and human-induced pluripotent stem cells are increasingly used to generate dopaminergic neurons and glial cells for microfluidic and OoC models of PD, offering a route toward personalized disease modeling (Table 1).

We combined the existing single-cell studies with OoC research and created a unified Table 1 describing the samples used, microfluidic technology and main findings in each study. Based on Table 1, we find several emerging key trends regarding the use of microfluidic systems in the study of PD.

First, all research efforts focus primarily on αSyn, although other proteins are known to be involved in the formation of Lewy bodies and neurites [14]. For example, Gribaudo et al. identified insoluble deposits of SQSTM1-p62 and HSP-70, the levels of which correlate with mitochondrial dysfunction [95].

Second, many studies suggest that mitochondrial dysfunction serves as an early indicator of PD, discussing impairments in processes such as mitophagy, calcium homeostasis, and anterograde axonal transport of mitochondria [16, 90, 95, 96].

Third, axonal transport is a promising avenue of research in synucleinopathies, as impaired trafficking of αSyn aggregates contributes to disease progression via prion-like spreading. Studies show that pathological αSyn fibrils undergo anterograde and retrograde transport along axons, facilitating neuron-to-neuron transmission and seeding endogenous aggregation [95, 97, 98]. Studies in the context of the prion-like mechanism of αSyn action showed trans-synaptic propagation of synuclein fibrils from the primary site along established neuronal pathways [17]. Almost all studies either support or do not contradict this hypothesis. Importantly, microfluidic technologies allow the study of internalized fibrillar structures as well as ribbon-like forms and oligomers [95–98]. These systems not only allow quantitative characterization of ongoing processes, but also effectively facilitate the separation of axons from the soma, which is essential for in-depth analysis [91].

There is an increasing interest in exploring the different conformational structures of αSyn [18]. As noted above, αSyn fibrils are not as toxic as oligomers and other intermediate structures [99], and oligomeric forms are more toxic in almost all neurodegenerative diseases. This also applies to proteins such as huntingtin, tau and amyloid β (Aβ) [100]. Proposed mechanisms of this toxicity include disruption of axonal transport, increased membrane permeability and disruption of calcium homeostasis [20, 21, 101]. However, a major challenge in studying these oligomeric forms is their high heterogeneity, which requires the development and application of specific detection and analysis methods.

## Sensors overview in PD

There are different sensor techniques used for LoC devices, including electrical sensors, electrochemical sensors, optical sensors, and immunological sensors, including enzyme-linked immunosorbent assay (ELISA) and immunofluorescence (IF) staining. Each method offers distinct advantages depending on the specific characteristics of the samples being analyzed [102]. One of the most commonly used detection methods is fluorescence, which belongs to the category of optical sensors. Fluorescence detectors are often preferred for their high sensitivity and specificity [56, 103]. This category also includes absorption spectroscopy and optical ring resonators, which detect changes in light as it passes through the sample [104, 105]. Additionally, impedance spectroscopy is a popular method that measures changes in the electrical resistance of a sample as it interacts with the microfluidic system. This technique allows for the assessment of various sample properties, such as conductivity and dielectric properties [106].

Although less commonly used, the colorimetric method remains an important detection tool [107, 108]. It relies on changes in the color of the sample resulting from chemical reactions, making it useful for assessing the concentration of specific substances. Electrochemical detectors, typically using three-electrode systems, can also be used for analysis and detection [107]. These detectors have the advantage of not requiring special dyes or fluorescent labels, reducing reliance on sample preparation and often proving to be more cost-effective [109]. In addition, mass spectrometry (MS) can accurately determine the molecular weight and structure of proteins. This method analyzes the charge-to-mass ratio of particles, allowing the chemical composition of compounds to be identified and their molecular structures to be assessed [110, 111]. In summary, selection of appropriate detection methods for a chip should be based on the type of sample being analyzed and the specific data required. Each method has its own strengths and limitations, and a combination of methods may provide the most comprehensive and accurate analysis.

### αSyn detection modalities in LoC systems

The choice of detection method in LoC systems is clearly determined by the nature of the samples being analyzed. In the context of αSyn research, the most commonly used methods are IF techniques based on the interaction between antigens and antibodies. Among these methods, ELISA and its various modifications, including sandwich immunoassays, stand out [112–114]. These techniques are well suited for integration with LoC systems, allowing effective detection of αSyn in a range of biological samples such as blood, cerebrospinal fluid (CSF) and skin [115–117]. However, it is important to note that ELISA and similar methods require careful selection of dyes and can be highly operator-dependent [118]. Specific dyes are chosen based on the structure, kinetics and source of the protein, which can complicate the analysis, particularly when dealing with αSyn oligomers. Traditionally, thioflavin T (ThT) has been used to stain amyloid fibrils [119]. However, this dye has insufficient specificity and sensitivity for αSyn, prompting the development of new molecules [119]. Notable among these new dyes are derivatives of benzothiazole and N-arylaminonaphthalene [120–122]. These novel dyes are designed not only to increase sensitivity and specificity, but also to ensure that their interaction with the protein does not induce conformational changes [120]. Other staining methods suitable for αSyn monomers and oligomers include the carbocyanine dye JC-1 [123]. In addition, universal dyes that can be used for all forms of αSyn have been proposed, such as Thioflavin X and tetraphenylethene-triphenylphosphonium (TPE-TPP) [124, 125]. In addition, TPE-TPP has the unique ability to selectively stain mitochondria that are damaged early in the development of PD, in addition to binding to αSyn aggregates [126].

Another important category of detection methods includes electrochemical techniques, such as impedance spectroscopy and various types of pulse voltammetry [127]. These electrochemical methods rely primarily on the use of aptamers to bind to target molecules such as αSyn. Aptamers are short, single-stranded DNA or RNA molecules capable of interacting specifically with certain targets, often referred to as ‘artificial antibodies’ [128]. The process of selecting these aptamers, known as Systematic Evolution of Ligands by Exponential Enrichment (SELEX), can be divided into three main stages. The first stage involves the binding of aptamers to the target molecule under optimal conditions. The second stage separates molecules that do not bind effectively from those that successfully interact with the target. The final step is to amplify the remaining molecules that have a high affinity for the target molecule [129, 130]. This cycle can be repeated several times to achieve the best results with maximum affinity. The SELEX process allows the selection of the most effective binding molecules from a large pool of random sequences, making it an essential tool for the development of specific detectors in electrochemical methods [129]. Aptamers offer several advantages, including non-immunogenicity, high thermal stability and, most importantly, the ability to discriminate between different conformations of the same protein [131].

**Fig. 4:**
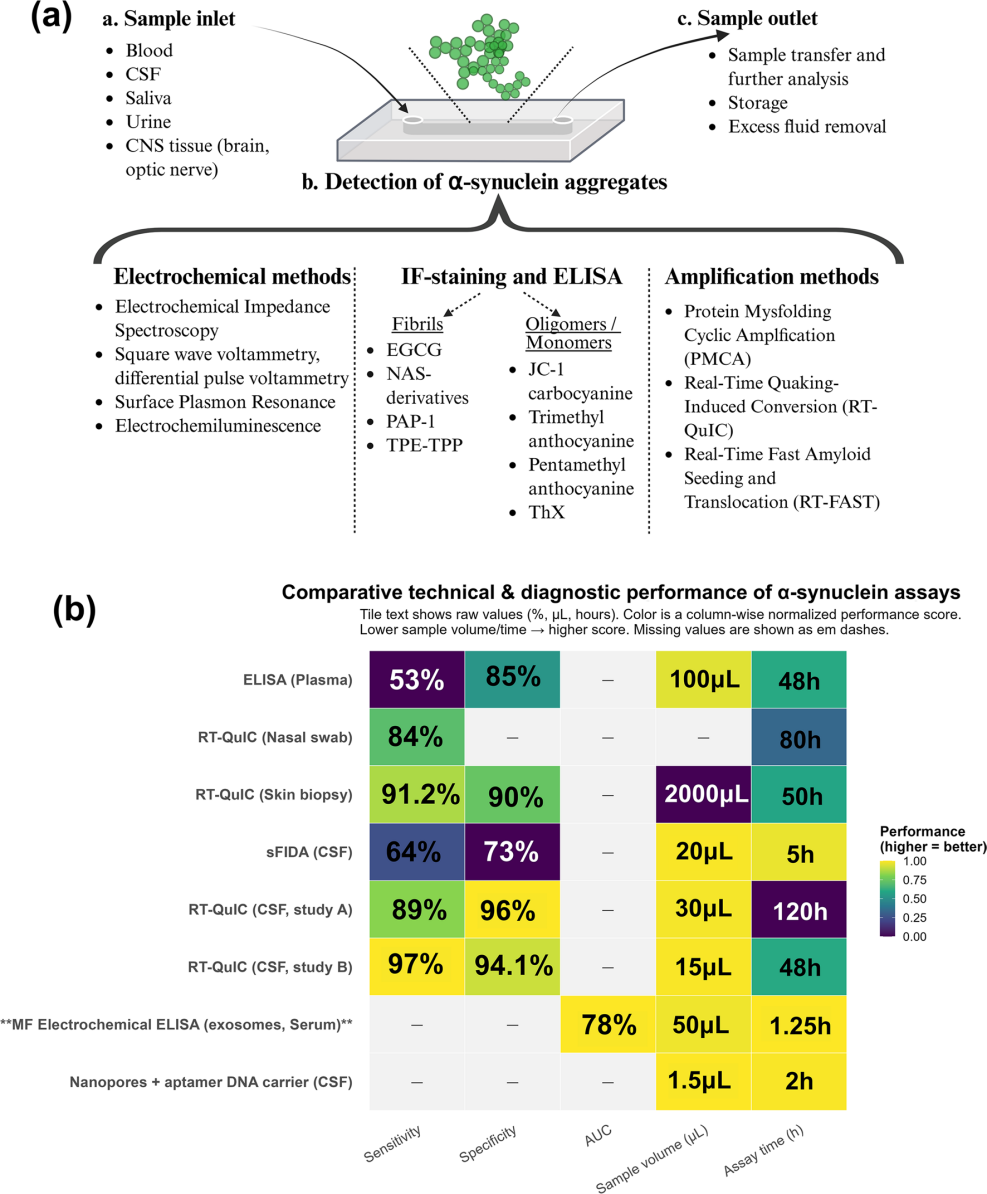
Methods for detecting αSyn in LoC devices. **a** LoC-based αSyn detection spans amplification (PMCA, RT-QuIC, Real-Time Fast Amyloid Seeding/Translocation) [24, 26, 237, 238, 242], dyes, and electrochemical aptamer biosensors [128]. Thioflavin T and Congo red flag fibrils may misread oligomers [119, 122]; newer NAS derivatives better target αSyn oligomers [121, 239]. EGCG promotes aggregation [240]; TPE-TPP helps visualize fibrils and mitochondrial damage [125, 126]. JC-1 and ThX (Thioflavin X) detect oligomers and monomers [123, 124]. Aptamer sensors enable rapid, minimally invasive (even saliva) and specific readouts, with therapeutic exploration ongoing [236, 241]. **b **A heatmap compares assays on sensitivity, specificity, AUC, sample volume, and assay time (0–1 column-normalized; darker = better). RT-QuIC (CSF) leads on sensitivity/specificity, ELISA (plasma) trades higher specificity for lower sensitivity, while microfluidic electrochemical ELISA (exosomes/serum) and nanopores + aptamer DNA carriers excel in low volume and speed [158, 217, 222, 243–247]. Tiles show raw values (%, μL, hours); lower volume/time and higher % score higher; missing values are em dashes. Generated in RStudio from the cited datasets. αSyn, α-synuclein; LoC, lab-on-a-chip; TPE-TPP, tetraphenylethene-triphenylphosphonium.

There are some other prime examples of sensors developed specifically for αSyn [45, 102, 132, 133]. Wu et al. developed electrochemiluminescent aptasensors capable of detecting oligomeric αSyn with femtomolarsensitivity in diluted serum, achieving detection limits of 0.42 and 0.38 fmol/L using two distinct functionalization protocols on indium tin oxide glass [45]. These sensors highlight the potential for ultra-sensitive detection in biofluids. Similarly, Lee et al. utilized peptide-imprinted polymers to measure soluble αSyn levels in supernatants from midbrain organoids [132]. Their study demonstrated reduced soluble αSyn in PD-specific organoids with a gene triplication mutation, a finding further corroborated by IF analysis [132]. Adampourezare et al. also reviewed the expanding applications of molecularly imprinted polymer-based sensors for neurodegenerative disease biomarkers, emphasizing their growing relevance in advancing biomarker detection strategies [133]. The detection methods for αSyn described above are illustrated in Fig. 4.

### Oligomer detection

The challenges in detection of αSyn and the wide variety of novel methods for detection may be based upon the fact that the oligomer of αSyn is not a strictly standardized molecule but exists in a variety of different conformations. Typically, these oligomers are classified as ring-shaped and spherical [31]. When αSyn is incubated under conditions conducive to fibril formation, such as in the presence of compounds like polyphenol(-)-epigallocatechallate, nicotine and 3,4-dihydroxyphenylacetic acid, the most common intermediate products are spherical conformations [32–35]. In addition, long tubular structures have been described, the formation of which is related to the duration of incubation [36]. The different oligomeric forms may differ in their toxicity and interactions with cells. For example, one study found that toxicity is only associated with compact oligomers that are resistant to proteinase K and form as a result of conversion from the parent molecules [37].

The formation of specific oligomeric forms is influenced not only by the availability of monomers but also by environmental conditions such as temperature and pH [38]. Point mutations in the *SNCA* gene, which encodes αSyn, can lead to early forms of PD by increasing the synthesis of monomers that serve as a substrate for oligomer formation [39]. In addition, different mutations can lead to the production of specific types of oligomers. For example, the A53T mutation promotes the formation of annular and tubular structures [36]. Therefore, researchers must consider all relevant conditions, including the microenvironment, as they will directly affect the results obtained from in vitro models.

Quantitative characterization of αSyn oligomeric molecules using traditional research methods is severely hampered by the low availability of molecules and their high heterogeneity. In this context, microfluidic technologies offer an effective approach for the detection of oligomeric molecules in cells due to their ability to interact with extremely small volumes of fluid and their integration with various detection methods, including dynamic studies [99, 100]. Numerous experimental studies have highlighted the importance of microfluidic technologies for the detection and quantitative characterization of oligomers. For example, a study in 2024 using microfluidic diffusion sizing (MDS) and μFFE showed that αSyn oligomer molecules have a 150-fold greater affinity for cellular membranes than the monomers. A notable advantage of MDS is its independence from changes in the secondary structure of the protein, making it particularly valuable for studying the highly variable forms of αSyn [18].

In another study, the combination of rapid-flow microfluidics with fluorescence analysis allowed real-time assessment of the oligomerization process. The authors identified different subgroups of oligomers and found that early forms are able to dissociate in low ionic strength solutions, which may facilitate further methods to isolate oligomeric molecules [99]. Another important platform is the multispectral microchip μFFE introduced by Arter et al. [134]. This tool allows the analysis of oligomeric structures under physiological conditions. The integration of this technique with microfluidic chips significantly accelerates the analysis process and demonstrates high colloidal stability of oligomers, which are relatively long-lived forms with a low tendency for direct aggregation [134].

It is important to note that αSyn oligomers can be found not only in brain tissue but also in CSF and blood, albeit at extremely low concentrations ranging from 1 zeptomolar to 1 picomolar [36]. The detection of these oligomeric molecules can serve as a reliable biomarker for the disease at the preclinical stage [61, 62]. Microfluidic technologies can be integrated at different stages of laboratory diagnostics to improve detection capabilities. For example, the study by Sierks et al. used microfluidic chambers as part of a biosensor that included a nanoporous aluminum membrane and electrochemical impedance spectroscopy (EIS) for result detection [46]. The authors emphasized the importance of selecting antibodies that recognize different forms of αSyn. For example, nanobody D10 can bind to any variant of αSyn, whereas antibody D5 is suitable for specific oligomeric forms. Notably, in this study, αSyn oligomers were detectable even at tenfold dilution, highlighting the high sensitivity of the technologies and biomarkers used [46].

Another interesting process is the co-oligomerization of αSyn with other proteins [65]. Both αSyn oligomers and monomers have been shown to interact with other proteins, including Aβ and tau protein [66]. However, co-oligomerization with tau protein has only been demonstrated under in vitro conditions [65]. In that paper, a microfluidic device was used to channel single molecules, followed by fluorescence-based detection, which significantly reduced the analysis time [65].

The use of antibodies to study oligomers presents significant challenges, although they have high detection potential. For example, a recent study tested 16 antibodies designed for conformation-specific recognition, but none demonstrated specificity for the size of αSyn aggregates [135]. As a result, techniques such as ELISA and various fluorescence studies are proving inadequate for the accurate identification of oligomers. They require integration with microfluidic analysis to effectively control the experimental environment. This need arises not only from poor understanding of the molecular structure of oligomers, but also from their ability to cross-react with both amyloidogenic and non-amyloidogenic proteins [136]. By addressing these complexities, researchers can improve the reliability of their findings and develop more effective diagnostic and therapeutic strategies for neurodegenerative diseases. The integration of advanced methods with microfluidic technology could pave the way for more precise and sensitive analyses, ultimately improving our understanding of αSyn and its role in the pathology of conditions such as PD.

### Fibril detection

The use of microfluidic technologies to study αSyn fibrils has become common in the context of investigating prion-like propagation of aggregates and their axonal transport [17]. While many researchers have used microfluidic techniques for various purposes, αSyn fibrils have consistently served as an intermediate focus of study. For example, one study investigated the disaggregation of αSyn fibrils under the influence of the chaperone HSP70, using microfluidic technologies in the microfluidic diffusional sizing method to determine the molecular weights of the molecules involved [137]. In addition, studies have directly applied microfluidic diffusional sizing to study the kinetics of αSyn aggregate growth using nanotubes [138]. Table 2 provides an overview of the major detection strategies for different αSyn species, spanning monomers, oligomers, protofibrils, and fibrils. In addition to summarizing conventional biochemical and biophysical methods, the table highlights contexts in which microfluidic platforms can be integrated to improve sensitivity, reduce sample volumes, or enable real-time monitoring. Key performance parameters such as detection limits, specificity, and typical sample requirements are also included, offering a comparative perspective on the strengths and limitations of each approach and where microfluidics may add unique value.

**Table 2:** Integration of microfluidic technologies in the characterization and detection of α-synuclein aggregation states

α-Synuclein type	Molecular weight range	Structural characteristics	Functional significance	Microfluidic applications and technologies	Detection methods in vitro	Detection limit	Typical sample volume	Specificity
Monomers	~ 15 kDa	Single, unstructured polypeptides	Non-toxic, baseline state	Single-Molecule Detection: Droplet Microfluidics for isolating individual monomers [219], μWestern Blot for protein quantification [262]; Real-Time Analysis: Fluorescence (ELISA) [259]	Spectroscopy: FCS, FRET [141]; MS Microscopy: TEM [120]; Electrophoresis: CE, SDS-PAGE [263]; μFFE [220] Single-Molecule Techniques: SMFS [144], Optical Tweezers [143]	• FCS: Low nmol/L–pmol/L; fibril ≥ 0.4 μmol/L[264] • FRET: 50 nmol/L (72 h); 500 nmol/L (3 h) [265]; oligomers 6.3 nmol/L [266]; fibrils 1 pM [267] • MRM-MS/MS: 4 ng/mg tissue [189]; 3–30 pg/mL CSF [177] • SDS-PAGE/WB: ~ 25 pg total protein [268] • CE-MS: 0.02 μg/mL [269] • sFIDA: 6.72 fM [244] • ELISA: 36.3 pg/mL [270]	• Microfluidics: 10–20 pL droplets (spans pL–nL); μFFE μL flow [134, 271] • NMR: 500–600 µL (5 mm tube) [272] • CD: ~ 10 µL [273] • FCS/FRET: fL probe; nL–µL chambers [274] • SDS-PAGE: 20–30 µL [275] • CE: nL injections [276] • TEM: 3 µL/grid [277] • AFM: 10 µL [278] • SEC: ~ 100 µM aliquot [279] • ThT: 20 µM final solute [273] • ELISA: 10 µL lysate/well [221]	• Conformation-sensitive (FCS/FRET) [274] • Proteoforms (MRM-MS/MS) [267, 177] • PTMs, Ser129-P (WB) [268] • Aptamer-based (CE-MS) [269] • Aggregates vs tau (sFIDA) [244] • Antibody-defined isoforms (ELISA) [270]
Oligomers (Dimers and Trimers)	~ 30–45 kDa	Small, soluble, initial aggregates	Early toxic species, rate-limiting step	Aggregation Monitoring: Microfluidic Mixing for rapid reagent combination [150], Single-Molecule Fluorescence for detection of small oligomers [219]	Spectroscopy: CD, FT-IR, NMR [142]; MS Microscopy: TEM, AFM [241, 252] Electrophoresis: SDS-PAGE [263], μFFE [220]; Single-Molecule Techniques: SMFS [142]			• FRET aptasensor selective for oligomers [265] • CD/NMR β-sheet transition [271] • TEM/AFM morphology (spherical/annular) [277]
Protofibrils	260 kDa and higher	Semi-structured, elongated chains	Highly toxic, membrane-disruptive	Interaction Studies: αSyn-membrane interaction studies [146], Microfluidic Flow Systems for simulating physiological conditions [89, 257]	Microscopy: TEM, AFM [280]; MS Chromatography: SEC [281]; Spectroscopy: EM, Raman [280, 282]	—		• SEC: separates protofibrils vs monomers/oligomers [279] • TEM/AFM elongated chains [277] • Raman β-sheet enrichment [273]
Fibrils	Insoluble	Highly structured, stable aggregates	Hallmark of PD	Structural Analysis: Thioflavin T Assay for high-sensitivity quantification, polymorphism studies [115]	Microscopy: TEM, AFM [280]; MS Spectroscopy: EM, SPR, Raman [280, 282]; Fluorescence Assays: ThT Assay [283], FRET [218], sFIDA [244]	—	—	• ThT: β-sheet fibrils [273] • FRET: seeding conversion [265] • sFIDA: fibrils vs soluble [244]

*AFM* Atomic force microscopy, *CD* Circular dichroism, *CE* Capillary electrophoresis, *CE*-*MS* Capillary electrophoresis–mass spectrometry, *CSF* Cerebrospinal fluid, *ELISA* Enzyme-linked immunosorbent assay, *EM* Electron microscopy, *FCS* Fluorescence correlation spectroscopy, *FRET *Förster (Fluorescence) resonance energy transfer, *FT-IR* Fourier transform infrared spectroscopy, *MRM-MS/MS* Multiple reaction monitoring tandem mass spectrometry, *MS* Mass spectrometry, *μFFE* Micro free-flow electrophoresis, *NMR* Nuclear magnetic resonance (Spectroscopy), *SEC* Size-exclusion chromatography, *sFIDA* Surface-based fluorescence intensity distribution analysis, *SMFS* Single-molecule force spectroscopy, *SPR* Surface plasmon resonance; *TEM* Transmission electron microscopy, *ThT* Thioflavin T, *μWestern blot* Micro-western blot, *PTMs* Post-translational modifications

Modern fluorescence imaging techniques often rely on the interaction with thioflavin T, which is particularly effective for the detection of fibrillar structures [139, 140]. However, some researchers emphasize the importance of using technologies that operate at the single-molecule level, such as fluorescence resonance energy transfer (FRET) and FCS [141, 142, 143, 144]. A major challenge with these methods is the difficulty in selecting antibodies that can specifically recognize the desired conformational states of the protein [145].

The methods used to detect monomers are similar to those used to identify oligomers, as they all focus on the manipulation of small molecules. Microfluidic technologies provide an ideal platform to carry out these manipulations, allowing the analysis of single cells. Studies using microfluidic approaches to analyze αSyn monomers and oligomers have focused on investigating the kinetics of aggregation [219, 146]. Particular emphasis has been placed on identifying the rate-limiting stage of aggregation and investigating the critical cores of oligomers, which has aided in the development of several aggregation inhibitors [39, 147, 148, 149]. Research into the bioredistribution of drugs also offers new opportunities to reduce the toxicity of oligomers [150]. These approaches are based on blocking the interaction of oligomers with cell membranes, effectively leading to their inactivation.

Current research often identifies oligomers and protofibrils as the primary toxic entities, with mature fibril aggregates considered either as a consequence of disease progression or even as a protective mechanism. However, some studies suggest that fibrillar structures may interact with plasma membranes, disrupt calcium homeostasis and cause mitochondrial dysfunction [151–153]. Furthermore, the presence of intracellular αSyn aggregates triggers microglial activation and the synthesis of key pro-inflammatory cytokines (TNF-α, IL-1β), leading to chronic inflammation [154]. In addition, αSyn fibrils have been shown to recruit free endogenous protein molecules, further promoting the formation of amyloid complexes [15]. Despite these findings, there is a lack of research directly comparing the toxic effects of fibrils and oligomers in living organisms, leaving the ongoing debate about the primary toxic conformation of αSyn unresolved. This knowledge gap highlights the need for more comprehensive studies that can elucidate the different roles and mechanisms of these conformations in the context of neurodegenerative diseases.

### Comparative overview of αSyn detection assays

Methods described in Table 2 are not exhaustive; in fact, one αSyn assay can contain a combination of both optical and immunological methods applied at different stages. In general, one could divide αSyn methods into quantitative and qualitative types, with the first type being of utmost interest in PD diagnostics. The methods from Table 2 could also be grouped into optical, electrochemical and immunological assays. We created Table 3 with different αSyn assays and their characteristics to compare between different methods of αSyn detection. As a result, vast majority of studies were focused on precise PD diagnosis using small amounts of samples. We also noted the need for differential diagnosis between different αSyn pathologies, where novel assays prove to be much weaker compared to regular disease/control comparison. RT-QuIC offers a highly sensitive and specific method for detecting misfolded αSyn, capable of producing rapid results (1–2 days) from small biological samples like CSF. Its scalability, quantitative ability, and applicability to early-stage diagnosis make it a promising tool for PD and related synucleinopathies [155]. For instance, RT-QuIC has demonstrated very high diagnostic accuracy, achieving over 90% sensitivity and specificity in distinguishing dementia with Lewy bodies (DLB) from controls and Alzheimer's disease, though differentiation between DLB and PD remains challenging [155]. Notably, a refined αSyn RT-QuIC assay was developed that combines rapid turnaround—reducing processing time from over a week to just 1–2 days—with strong diagnostic performance. This assay can sensitively detect and quantify disease-associated αSyn seeding activity in minute CSF samples, even at early symptomatic stages of synucleinopathy [156].

**Table 3:** Comparative overview of optical, electrical, and immunological methods for αSyn detection from clinical and technical perspectives

Method (category)	Sample(s)	Clinical use focus	Reported diagnostic performance	Technical considerations (assay time, equipment, workflow)	References
Optical—CSF αSyn seed amplification (RT-QuIC / SAA; ThT fluorescence)	CSF	Differential diagnosis of Lewy body disease: strong for DLB vs controls/AD; supportive for PD; less useful for PD vs DLB	Meta-analysis in DLB: pooled sensitivity 94% / specificity 96% (vs controls) and 95% / 88% (vs AD), discrimination of DLB vs PD remains poor (sensitivity ~ 94% but specificity ~ 11%)	Standardized CSF preanalytics (15–20 mL, centrifuged, aliquoted, − 80 °C). 96-well plate with silica beads, recombinant αSyn substrate + ThT, read at 42 °C with shake–rest cycles. Quadruplicate wells, threshold = baseline + 3 SD. Time-to-result ~ 2–4 days	[156, 155]
Optical—Nasal mucosa αSyn SAA (RT-QuIC)	Olfactory mucosa swab	Ancillary test to PD diagnosis when CSF undesirable	Agger nasi sampling improved sensitivity to ~ 84% vs ~ 45% from middle turbinate; CSF same cohort 94% sensitivity	Non-invasive nasal swab; 2–4 samples collected, well tolerated. Pellets processed by sonication, seeded into 96-well RT-QuIC with recombinant αSyn substrate and ThT. Assay run at 30 °C with 1-min shake / 14-min rest cycles, read every 45 min, cutoff at 80 h. Requires only ENT swab and plate reader, offering patient-friendly alternative to lumbar puncture	[158]
Immunological—Skin biopsy pSer129 αSyn (IHC/IF)	3 mm punches (distal leg, distal thigh, posterior neck)	In-vivo pathology confirmation across synucleinopathies; office-based tissue biomarker	95.5% overall (213/223 synucleinopathy); PD 92.7% (89/96)	Well tolerated; minor bleeding 0.5%; no serious adverse events; high concordance; office-based; rapid, reproducible; needs histopathology	[117]
Optical + Immunological—Serum IP/RT-QuIC	Serum	Blood-based pathology readout; strong for PD/DLB vs controls; lower for MSA	PD diagnostic performance: AUC 96% (cohort 1), 93% (cohort 2), 86% (external cohort) vs controls	Adds IP enrichment to RT-QuIC; authors note substrate lot criticality and seed-concentration dependence; runtime similar to RT-QuIC; standard plate reader + IP workflow	[157]
Immunological—Neuronally derived EV αSyn (ECL immunoassay)	Serum / plasma EVs	Risk stratification / prodromal screening for PD	Multi-cohort (*n* = 576): AUC 90% distinguishing ≥ 80% prodromal PD-probability vs controls	Multicohort validation with reproducible thresholds; serum EV-based, minimally invasive; correlates with prodromal markers, DaT SPECT, and CSF SAA; robust across labs; limited by cross-sectional design, specialized EV isolation needs, and lack of automation	[162]
Optical—sFIDA (single-particle fluorescence counting)	CSF, stool, urine	Aggregate-focused readout; exploratory as a clinical aid (PD/DLB), potential for prodromal and non-CSF matrices	PD vs controls: specificity 73%, sensitivity 64%, AUC 68%	sFIDA detects CSF αSyn aggregates with femtomolar sensitivity, high reproducibility, negligible cross-reactivity, and confirmed specificity; requires TIRF microscopy and nanoparticle calibration	[223]
Optical / Biophysical—FCS, smFRET	In-vitro samples / research	Mechanistic profiling (early oligomers, kinetics); not for routine diagnosis	Tracks early oligomer formation and conformational changes in real time; demonstrates contagious conformational change during early aggregation	Confocal/single-molecule setups; fL volumes; expert-only; useful to validate targets/conformations, not clinical labs	[274]
Electrical—Microfluidics EGOFET biosensor	Buffer (proof-of-concept; future: CSF/serum)	Prototype POC tool for αSyn detection; potential diagnostic application for PD and other synucleinopathies	Detection range: 0.25 pmol/L–25 nmol/L; LOD = 0.25 pmol/L	Label-free, antibody-functionalized gate; coplanar microfluidic design prevents cross-contamination; assay time minutes; compatible with compact, scalable chip manufacturing	[116]
Electrical – Soft microfluidics OEGFET aptasensor	Saliva	Non-invasive point-of-care detection of αSyn monomer; potential PD screening in aging populations	LOD = 10 fg/L; linear range 100 fg/L–10 μg/L; covers physiological saliva αSyn monomer levels	Label-free; aptamer selective to αSyn monomer; reusable sensor; fully integrated soft microfluidic channels for simple saliva handling; isolated organic semiconductor extends shelf life; assay fast and minimally invasive	[267, 177]

*CSF* cerebrospinal fluid; *EVs* extracellular vesicles; *DLB* dementia with Lewy bodies; *MSA* multiple system atrophy; *AD* Alzheimer’s disease; *ThT* thioflavin T; *SD* standard deviation; *ENT* ear, nose, and throat; *AUC* area under curve; *RT-QuIC* real-time quaking-induced conversion; *ECL* electrochemiluminescence; *TIRF* total internal reflection fluorescence; *FCS* fluorescence correlation spectroscopy; *smFRET* single-molecule fluorescence (or Förster) resonance energy transfer; *EGOFET* electrolyte-gated organic field-effect transistors; *POC* point-of-care; *LOD* limit of detection; OEGFET organic electrolyte-gated field-effect transistor; *αSyn* α-synuclein

Optical methods such as seed amplification assays (SAAs) (e.g., RT-QuIC) are highly sensitive and specific, allowing detection and quantification of misfolded αSyn aggregates through their amyloid seeding activity, often enabling early and rapid diagnosis of synucleinopathies like PD [155, 156, 157, 158]. In contrast, immunological methods like ELISA rely on antibodies to measure total or modified αSyn levels in biofluids, but their sensitivity can vary depending on the assay platform and antibody specificity, and they may not fully capture all disease-relevant αSyn forms [159, 160]. Immunological assays are widely used due to their practicality and established protocols but can be affected by factors such as protein modifications or sample handling, sometimes resulting in overlapping signals between patients and controls [112, 159–161]. While immunological assays such as ELISA are widely used for measuring total αSyn levels in plasma or CSF, they often fail to detect disease-relevant proteoforms such as truncated or post-translationally modified αSyn, which can lead to variability in diagnostic accuracy. For example, newer high-sensitivity platforms like Quanterix Single Molecule Array show improved effect sizes but still cannot fully capture the complexity of αSyn species present in synucleinopathies [160, 161].

However, immunological methods have been applied to detect αSyn in skin biopsies, which are easier to obtain than CSF typically used in SAA. For instance, in a large multicenter 2024 study involving over 300 participants, phosphorylated αSyn was detected in more than 90% of patients with PD, dementia with Lewy bodies, multiple system atrophy, and pure autonomic failure, whereas only 3.3% of controls tested positive [117]. This study demonstrated not only high sensitivity and specificity but also the potential clinical utility of skin biopsy as a minimally invasive diagnostic tool that can influence diagnosis and treatment decisions in synucleinopathies. Immunological studies have also been modified to detect αSyn in serum, a more easily obtainable sample compared to CSF. A 2024 multicenter study analyzed extracellular vesicles (EVs), specifically neuronally derived serum EVs (NDEVs) (L1CAM-positive EVs) containing αSyn, and found that this biomarker distinguished individuals with isolated rapid eye movement sleep behavior disorder (iRBD)—a prodromal phase of PD—from controls with an area under curve (AUC) of 0.91. Moreover, αSyn levels in these vesicles identified subjects with more than 80% probability of prodromal PD with an AUC of 0.80 and predicted phenoconversion to PD or related dementia in 80% of cases [162].

Taken together, the assays summarized in Table 3 show that αSyn detection has reached impressive levels of analytical sensitivity and diagnostic specificity, particularly in CSF- and biopsy-based studies. Nevertheless, their broader clinical translation is still limited by several practical constraints, including invasive sampling procedures such as lumbar puncture, long and technically demanding workflows that may extend up to several days, and the requirement for specialized instrumentation and centralized laboratory settings [117, 155, 156, 157, 158, 162]. In contrast, the emerging microfluidic approaches listed in Table 3 illustrate how miniaturized platforms can overcome these barriers. By integrating fluid handling directly on-chip, microfluidic devices sharply reduce sample and reagent consumption (e.g., saliva-based OEGFET [Organic Electrolyte-Gated Field-Effect transistor] aptasensors with detection in the fg/L range), simplify workflows into compact and reusable formats, and enable rapid, label-free analysis in minutes rather than days [116]. Moreover, microfluidics provide a versatile framework that can incorporate immunological, optical, or electrochemical transduction within the same device, paving the way toward scalable, non-invasive, and point-of-care αSyn diagnostics [163]. Taken together, these developments highlight how microfluidic platforms could make αSyn testing faster, less invasive, and more practical outside of specialized labs. At the same time, an equally important question is where the protein can best be measured. Recent studies have begun to focus on the different tissues and fluids in which αSyn appears, especially those that may show changes early in the course of disease.

## Substrates for αSyn detection

αSyn is being investigated as a potential biomarker for PD, as its accumulation in peripheral tissues may precede the onset of classic motor symptoms of the disease [164, 165]. Prior to the onset of typical motor symptoms, patients often experience dysfunction in various organs and systems that may be related to the accumulation of this protein. Early signs such as urinary dysfunction, constipation, depression and anosmia may indicate the early stages of the disease [166, 167]. Research is actively exploring the possibility of isolating αSyn from peripheral tissues for early diagnosis. Particular attention is being paid to its secretion into biofluids such as CSF, blood and saliva [167]. In addition, αSyn can be detected directly in tissues such as the gut, salivary glands, peripheral skin nerves and various nerve plexuses in internal organs, including the heart [168–170].

According to recent studies and meta-analyses, the overall level of αSyn in the CSF is lower in patients with PD compared to control groups that include both healthy individuals and patients with Alzheimer's disease [112, 170–174]. However, significant differences in αSyn levels between PD and other synucleinopathies, such as dementia with Lewy bodies and multiple system atrophy, are often not observed [170–172]. Although the precise biological mechanisms underlying the reduced levels of αSyn remain poorly understood, several hypotheses have been proposed. One hypothesis is that αSyn is sequestered in Lewy bodies in the brain, while another is that its uptake by neurons is increased [175].

A 2015 meta-analysis found no clear correlation between CSF α-synuclein levels and disease severity, although some studies suggest that lower levels may be associated with more rapidly progressing phenotypes of PD [172, 176]. This meta-analysis also assessed the diagnostic value of CSF as a substrate for measuring αSyn. The sensitivity of the method (87%), mainly using ELISA, was found to be high but the specificity (42%) was low [172]. In a later meta-analysis in 2023, which looked at amplification techniques such as PMCA and RT-QuIC using microfluidic technologies, the specificity reached 0.95 compared with the control group, while the sensitivity was 0.88. However, both sensitivity and specificity decreased significantly in the differential diagnosis of various synucleinopathies [174]. In a comparative study, the specificity of the method for CSF as a substrate varied from 25% to 64%, while the sensitivity ranged from 61% to 94% [112]. Interestingly, despite reduction in total αSyn levels, the levels of its oligomeric and phosphorylated forms were often elevated, which may play a crucial role in understanding the pathogenesis of the disease and developing new diagnostic methods [170].

An alternative biological substrate for the detection of αSyn may be blood, including plasma, serum and erythrocytes [178, 179]. In addition to total αSyn, blood also contains one of its post-translational modifications—pS129—which is of particular interest because this form is present in Lewy bodies [180, 181]. The phosphorylated form and the oligomeric variant not only facilitate differential diagnosis but also correlate with disease severity; however, oligomeric αSyn only predicts prognosis when analyzed in the CSF [180, 182]. Numerous studies have confirmed elevated levels of oligomeric αSyn in the blood of PD patients compared with controls [183–186]. It has also been observed that blood levels of αSyn increase over the course of the disease [185]. However, as with total αSyn, the reference levels for the oligomeric protein have not yet been established and vary depending on the method and antibodies used for immunodetection. For example, a 2006 study using the ELISA method reported a level of 79.9 ± 4.0 pg/mL in the plasma of patients with PD [183]. In contrast, a 2017 study using a different kit found significantly different levels of αSyn, around 319.56 ± 64.22 pg/mL. In addition, the αSyn levels vary depending on the predominant PD phenotype of the patients [184].

A persistent obstacle in measuring αSyn in plasma or CSF is contamination from lysed red blood cells (RBCs), which harbor over 99% of blood αSyn [187]. Even slight hemolysis during sample collection or processing can markedly elevate fluid αSyn levels, distorting biomarker readouts in PD research [188]. While a few microfluidic blood–plasma separation devices report reduced hemoglobin (Hb) levels as a proxy indicator, such metrics do not correct for αSyn that is released into the sample during sample collection [187]. This raises a dual-edged concern: the high sensitivity of LoC platforms may amplify contamination, detecting trace αSyn derived from RBCs, which could generate misleading signals. However, LoC technologies offer unique advantages: they can integrate on-chip quality control measures such as Hb detection to flag contaminated samples, allow selective enrichment of neuronal αSyn-containing EVs less affected by peripheral blood contamination—such as through the use of L1CAM-positive EV immunocapture systems [179]—and support closed-system microfluidic workflows that minimize handling and reduce hemolysis risk right at the sample intake. Together, these features underscore that while LoC sensitivity poses challenges, its microfluidic precision and compartmentalization may ultimately help address one of the most persistent pre-analytical confounds in developing reliable blood-based αSyn biomarkers for PD.

When hemolysis is detected, a pragmatic correction path is possible. First, Hb thresholds are used to classify or exclude contaminated samples. For CSF, Paciotti et al. showed a near-linear rise of CSF αSyn with increasing blood contamination and recommended accepting only samples with Hb concentration < 1569 µg/L to limit inflation to < 5% [190]. For research analyses where mild hemolysis cannot be avoided, αSyn can be statistically adjusted using Hb as a covariate—the rationale is that RBCs are the dominant source of blood αSyn (more than 99%), so the hemolysis-dependent component can be modeled and subtracted. In addition, normalization strategies help reduce bias from residual hemolysis: β-synuclein (βSyn) is reported to be minimally affected by blood contamination, and αSyn/βSyn ratios or inclusion of βSyn as a normalizer can stabilize readouts [177]. Finally, proteomic work has defined multi-marker contamination indices that combine Hb with additional RBC-derived peptides, enabling detection of very-low-level blood contamination (down to 0.001%) that visual checks miss [191].

The detection of specific forms of αSyn, such as oligomeric, phosphorylated, and nitrated variants (the latter found exclusively in blood), necessitates the development of precise methods and highly sensitive antibodies capable of working with minimal substrate quantities [165]. To address this, innovative diagnostic techniques are continually emerging. For instance, a method introduced in 2022 combines a paired surface plasma wave biosensor with an immunoassay to analyze diluted human serum, achieving an impressive AUC of 0.92 [192]. While these advancements highlight the diagnostic potential of new technologies, the primary application of microfluidic systems remains in modeling pathology and constructing controlled microchambers to mimic disease conditions [193]. Nevertheless, microfluidic approaches have shown promise in diagnostics, with studies demonstrating the use of electrochemical impedance spectroscopy to analyze αSyn aggregates in saliva and blood [194], aptamer-based platforms for saliva analysis [195], and paper-based microfluidic devices for detecting oligomers in urine [196].

Early diagnosis of PD remains difficult because routine clinical evaluation and imaging typically identify disease only after substantial nigrostriatal degeneration occurs. Classic and contemporary reviews estimate motor onset occurs after large losses of nigral dopaminergic neurons and striatal dopamine, underscoring the need for fluid biomarkers detectable before overt symptoms [2, 6, 197]. LoC platforms address this by enabling high-sensitivity assays in minimal sample volumes via integrated microchannels and transducers, allowing detection of disease-relevant αSyn species from easily accessible biofluids (e.g., saliva, plasma) at concentrations below the reach of conventional ELISAs. For example, a soft-fluidic OEG-FET aptasensor quantified salivary αSyn with a limit of detection of 10 fg/L and a broad linear range, directly supporting non-invasive early screening in PD [195]. In parallel, SAAs—RT-QuIC/PMCA—already achieve high diagnostic accuracy for PD in CSF and other biospecimens and are being miniaturized/partitioned into microcompartments (digital SAAs), a strategy compatible with microfluidic implementation that improves precision and lowers the effective detection threshold for early, low-abundance seeds [115, 174, 198]. These microfluidic/LoC-amenable SAA formats are explicitly motivated by earlier, reliable detection in prodromal phases and have shown that protocol and partitioning choices significantly reduce measurement error—key for clinical deployment [199]. Massey et al. (2024) also proposed a surface-imprinted polymer (SIP) EIS sensor targeting αSyn with a low-cost, small-footprint design [194]. This SIP‑EIS biosensor achieved a detection limit of 5 pg/L, with detection range spanning 5 pg/L to 5 µg/L, making it highly suited for non-invasive, continuous monitoring via saliva or blood analysis. Lastly, Saadati et al. (2024) introduced a paper-based electrochemical immunosensor, fabricated using conductive nano-ink and biotinylated antibodies, capable of detecting αSyn antigen as low as 0.002 ng/mL, offering a portable and affordable point-of-care (POC) system for early PD screening [200]. Collectively, these LoC platforms improve upon current diagnostics by enabling earlier, easier, and more accurate detection of αSyn compared to conventional CSF or clinical motor-based assessments.

**Fig. 5:**
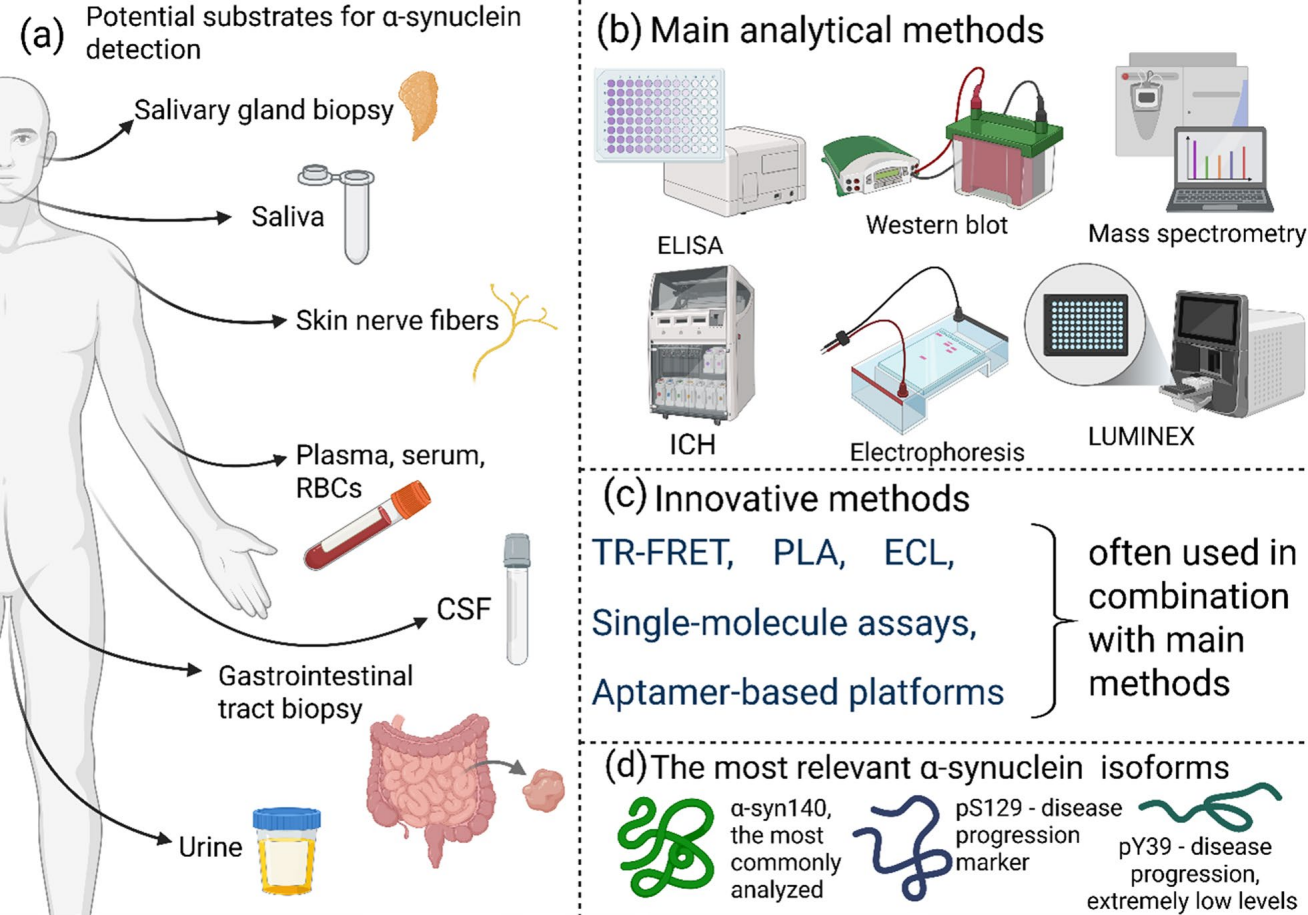
Potential substrates and analytical methods for αSyn detection. **a** αSyn, a protein implicated in the pathogenesis of neurodegenerative diseases such as PD, can be found in a variety of biological substrates, including cerebrospinal fluid (CSF), blood, urine, gastrointestinal tract and skin biopsies, which facilitate the analysis of aggregations at nerve endings [170, 183, 248–251, 253]. In addition, saliva and salivary gland tissue can be used for diagnosis. **b** The main analytical methods for αSyn include ELISA, Western blot, MS, ICH, electrophoresis and LUMINEX [172, 183, 184, 251, 253, 254, 263]. **c** Innovative methods such as Time-Resolved Fluorescence Resonance Energy Transfer (TR-FRET), electrochemiluminescence (ECL), proximity ligation assay (PLA), various single-molecule assays and aptamer-based platforms, are being used, often in combination with different analytical approaches, to achieve more accurate identification of this protein and its various forms [217, 195, 224, 254–256]. The choice of method largely depends on the form of αSyn being studied. **d** Although there are numerous isoforms of this protein, the aSyn140 isoform is the most commonly analyzed, together with total αSyn, oligomeric forms and their post-translational modifications [180, 224]. Among the most important modifications is the phosphorylated form pS129, which can be analyzed in both CSF and blood and is considered a potential marker of disease progression [192, 255]. Another notable modification is pY39, which is found at extremely low levels in biological substrates, necessitating the use of microfluidic technologies for its detection [180]

These studies underscore the unique advantages of microfluidic technologies for diagnosing PD and exploring novel substrates such as the gastrointestinal tract and skin biopsies, as schematically illustrated in Fig. 5 [201].

### Future directions and research priorities for LoC and OoC in PD

Looking forward, several high-impact avenues could shape the next wave of microfluidic and OoC technology for PD diagnosis and research.

#### Standardized, multiplexed biomarker detection

The heterogeneity of αSyn species (monomeric, oligomeric, phosphorylated) and overlap with other neurodegenerative biomarkers make multiplexing highly desirable. Recent general advances in microfluidic multiplex platforms—such as encoded microparticles enabling fast, simultaneous detection of several disease-related biomarkers—demonstrate technical feasibility and have translational potential in PD contexts [202]. Though not yet applied directly to PD, such systems could be adapted to detect αSyn alongside tau, amyloid‑β, or neurofilament light chain for improved diagnostic specificity.

#### Personalized therapeutic testing via iPSC-based systems

Although direct integration with microfluidics is still emerging, iPSC-derived neuronal models have demonstrated disease-specific phenotypes relevant to PD. For example, 3D microfluidic bioreactors have successfully differentiated human neuroepithelial stem cells into functional dopaminergic neurons—key to modeling PD in vitro [203]. Furthermore, reviews such as that by Fanizza et al. (2022) have underscored the promise of iPSC‑based OoC models for drug screening in neurodegenerative disorders, which could be tailored to PD in future studies [69].

#### Longitudinal, point-of-care monitoring

POC LoC devices present a unique opportunity for repeated, minimally invasive biomarker measurement. For instance, Massey et al. (2023) have demonstrated an OEG‑FET aptasensor capable of quantifying salivary αSyn aggregation, suggesting potential for real-world, non‑invasive monitoring [195]. Similarly, Saadati et al. (2024) presented a portable, paper‑based electrochemical immunosensor for αSyn in human plasma, highlighting feasibility of low‑cost POC platforms [200].

## Conclusions

Microfluidic platforms increasingly show promise for detecting αSyn aggregates at extremely low concentrations—an ability that is crucial for earlier and more accurate diagnosis of PD. Recent seed-amplification advances, including microstructured and compartmentalized formats, demonstrate that assay design can substantially improve sensitivity and turn-around time for misfolded αSyn detection and related proteopathic seeds [204–206].

Immediate short-term gains are achievable by standardizing substrate quality and reagents in SAAs (buffer composition, temperature/shaking parameters, and especially the choice and purity of recombinant substrate), while tightening pre-analytical controls for biofluids used on-chip. These factors are known to materially alter SAA kinetics and performance; systematic optimization improves the sensitivity/specificity and reproducibility across labs [207]. In parallel, sample quality should be guarded through clear handling standard operating procedures (e.g., hemolysis minimization for blood; controlled CSF collection/storage), since pre-analytical instability can bias the total and oligomeric αSyn measurements [208]. On the device side, surface chemistry to limit nonspecific protein adsorption (e.g., PEGylation, zwitterionic or protein blocking layers) and better channel designs to mitigate evaporation and bubble formation are practical microfluidic upgrades that raise analytical reliability without changing targets or readouts [209, 210].

Beyond shaking/sonication, field-assisted amplification is a promising direction for mid-term improvements: computational and experimental work shows that external electric fields can remodel or even disrupt αSyn fibrils, implying a route to tune nucleation/fragmentation dynamics; similarly, rotating magnetic fields modulate amyloid aggregation in related systems, suggesting a broader physical-control principle that could translate to αSyn SAAs [211, 212]. Alongside these concepts, microfluidic capillary-based QuIC and acoustofluidic Micro-QuIC have already demonstrated how altering the physical driving forces within the reaction environment can simplify hardware and accelerate amplification, respectively—blueprints for field-assisted SAA optimization within LoC devices [205, 206].

To move from sensitive detection to PD-specific diagnosis in the long run, emerging evidence points to EVs, especially NDEVs, as carriers of aberrant αSyn species that are less confounded by erythrocyte-derived α-syn. Multiple studies show plasma/serum NDEVs (often captured via L1CAM) harbor αSyn species that differentiate PD, iRBD, and high-risk cohorts, supporting EV-based blood tests for prodromal PD [162, 213]. Technically, next-generation EV workflows quantify whether αSyn resides inside EVs and can even enrich phosphorylated αSyn relative to bulk plasma, providing a finer-grained molecular readout for LoC integration [215]. Beyond EVs, peripheral immune compartments merit exploration: PBMCs and leukocyte-derived signals (including αSyn content and immune responses to αSyn) have been investigated as peripheral markers in PD, though findings are heterogeneous—an area where microfluidic single-cell or immunocapture methods could standardize and clarify leukocyte-based readouts [214, 216].

In summary, LoC technologies offer a bridge between early analytic feasibility and long-term diagnostic specificity and clinical translation in PD. Short-term efforts should concentrate on refining microfluidic robustness and multiplexed detection. At the same time, long-term progress hinges on rigorous clinical validation and integration with physiologically relevant OoC models to realize the full potential of PD-specific diagnostics.

## Data Availability

Not applicable.

## Abbreviations

*PD*: Parkinson’s disease
*αSyn*: α-Synuclein
*βSyn*: β-Synuclein
*LoC*: Lab-on-a-chip
*OoC*: Organ-on-a-chip
*iPSCs*: Induced pluripotent stem cells
*hiPSCs*: Human-induced pluripotent stem cells
*mDA*: Midbrain dopamine
*SC-RNAseq*: Single-cell RNA sequencing
*HD*: Huntington's disease
*Aβ*: Amyloid-β
*MDS*: Microfluidic diffusion sizing
*μFFE*: Micro free-flow electrophoresis
*μTAS*: Micro total analysis systems
*EIS*: Electrochemical impedance spectroscopy
*ELISA*: Enzyme-linked immunosorbent assay
*TEM*: Transmission electron microscopy
*EM*: Electron microscopy
*ThT*: Thioflavin T
*TPE-TPP*: Tetraphenylethene-triphenylphosphonium
*FRET*: Förster resonance energy transfer
*EVs*: Extracellular vesicles
*NDEVs*: Neuronally derived extracellular vesicles
*iRBD*: Isolated rapid eye movement sleep behavior disorder
*AUC*: Area under curve
*HNE*: 4-Hydroxy-2-nonenal
*BBB*: Blood–brain barrier
*BG*: Basal ganglia
*FCS*: Fluorescence correlation spectroscopy
*SELEX*: Systematic evolution of ligands by exponential enrichment
*CSF*: Cerebrospinal fluid
*PMCA*: Protein misfolding cyclic amplification
*RT-QuIC*: Real-time quaking-induced conversion
*PLA*: Proximity ligation assay
*IF*: Immunofluorescence
*MS*: Mass spectrometry
*SAAs*: Seed amplification assays
*PTMs*: Post-translational modifications
*DLB*: Dementia with lewy bodies
*Hb*: Hemoglobin
*RBCs*: Red blood cells
*POC*: Point-of-care
